# Supplementary material for: Intravitreal photoswitch therapy in advanced retinitis pigmentosa: a phase 1 open-label trial
Source: Nat Med. 2026 Apr 14;32(5):1865–70. doi: 10.1038/s41591-026-04317-6 (PMC13190265; doi:10.1038/s41591-026-04317-6)
Supplement: Supplementary file 1 — Supplementary Methods and caption for video file. [file 41591_2026_4317_MOESM1_ESM.pdf]

nature medicine

Supplementary information https://doi.org/10.1038/s41591-026-04317-6

Intravitreal photoswitch therapy in advanced retinitis pigmentosa: a phase 1 open-label trial

In the format provided by the authors and unedited

Supplementary Methods

Inclusion and exclusion criteria

Inclusion criteria

Participants were eligible if they met all the following criteria:

- Participants were adults aged 18–80 years of any sex or race.
- Participants were willing and able to provide written informed consent and to comply with study procedures and scheduled visits.
- Participants had a clinical diagnosis of retinitis pigmentosa.
- Participants had severe visual impairment meeting cohort-specific thresholds determined using the Berkeley Rudimentary Vision Test (BRVT):
  - Cohort 1: no light perception (NLP) or bare light perception (BLP) with logarithm of the minimum angle of resolution (logMAR) > 2.9.
  - Cohort 2: hand motion (HM) or counting fingers (CF) vision with logMAR ≤ 2.9 and > 1.6.
- Visual acuity was similar in both eyes, defined as an inter-eye BRVT logMAR difference of < 0.05.
- Participants agreed to avoid disallowed medications for the duration of the study.
- Participants of childbearing potential had a negative pregnancy test at baseline (where applicable) and agreed to use an acceptable method of contraception for the duration of the study.

Exclusion criteria

Participants were excluded if any of the following criteria were met:

- Participants had a clinically significant ocular or systemic condition that, in the investigator's judgment, could compromise safety or interfere with study participation.
- Participants had active ocular infection or inflammation.
- Participants had a history of retinal detachment.
- Participants were receiving prohibited concomitant medications.
- Participants had any condition that would prevent adherence to study procedures or follow-up.

Visual and functional assessments

Light perception assessment

This assessment involves showing the subject a series of videos and recording whether or not the subject perceived the visual stimulus, which was always an 'X' located at various locations on a screen. A strip of tape in front of the screen marked 60 cm where the center of the chair was placed and the subject seated. This assessment is meant to be performed as a monocular test and, therefore, an eyepatch was used to occlude the eye not being tested while assessing the contralateral eye. A unique video file driving the display of Xs on the screen was produced to correspond for each visit. Stimuli were presented using a calibrated display system, with three luminance settings corresponding to estimated photon fluxes of 3×10^{14}, 1×10^{15}, and 5×10^{15} photons·cm^{-2}·s^{-1} (400–800 nm). For each video, a series of six (6) images are displayed to the subject and the subject is

instructed to verbally indicate if they perceive a particular shape or object and the responses are recorded as a success or failure.

Visual acuity

Visual acuity (VA) was measured prior to pupillary dilation, both monocularly and then binocularly and testing was performed under normal room illumination (between 250 and 1000 lux). VA assessment was performed using the Berkeley Rudimentary Vision Test (BRVT; Precision Vision, Woodstock, IL) which consists of presenting subjects three hinged card-pairs:

- Single Tumbling E Card-Pair (STE)
- Grating Acuity Card-Pair (GA)
- Basic Vision Card-Pair (BV)

A testing flow chart, based on the ability of subjects to perform the required task, converts responses to a logarithm of minimum angle of resolution (logMAR) score. If the BV card-pair was not seen by the participant, light perception was assessed at 25 cm using a pen torch, and the resultant VA was either bare light perception (BLP) if seen, or no light perception (NLP) if not seen.

Kinetic visual fields

Kinetic visual fields were assessed using manual Goldmann perimetry with a blue light stimulus (440–460 nm). Testing was performed monocularly using standardised protocols. Visual field extent was quantified as the summed horizontal field extent across tested meridians.

Functional vision assessments (Ora-MLOM™)

The Ora-MLOM™ (Multiluminance Orientation and Mobility) system comprises a modular set of navigation and localization tasks performed under controlled ambient illumination conditions.

Tasks were administered at one or more predefined light levels (45 lux [low], 125 lux [mid], and 350 lux [high]), selected to span the operational range of the system. For the purposes of this exploratory study, different tasks were assessed at different illumination levels according to their dynamic range and discriminative capacity. Accordingly, walking direction performance was summarized across all tested illumination levels, whereas window and door location tasks were analyzed at specific illumination levels at which performance variability was most informative.

- The Window Location test is a stationary test that requires the subject to locate a randomly placed (front, left, centre, right, no window) white "window" approximately three feet by two feet attached to a black opaque screen for contrast. Each test is a sequence of 8 trials conducted at each lux level.
- The Walking Direction test is a stationary test that requires the subject to identify the direction of motion (to the left, to the right, or no motion) of a person walking between 2 opaque screens located 10 feet in front of them. Each test is a sequence of 6 trials conducted at each lux level.
- The Door Location test is a straight-line mobility course that requires a participant to locate and navigate to a randomly located (left, right centre) high-contrast door-like object placed on a wall 12 feet in front of them. Each test is a sequence of 6 trials conducted at each lux level.
- The Exit Room Test is a straight-line mobility test that requires a participant to follow a pathway while avoiding three randomly placed high contrast 2 x 2 feet foam obstacles. Each test is a sequence of 6 trials conducted at each lux level. Success requires the participant to navigate the course from start to end without (i) hitting 2 or more obstacles

during a single run, (ii) stepping completely off the white path with both feet or (iii) exceeding 3 minutes.

Functional magnetic resonance imaging (fMRI)

fMRI was performed to assess cortical responses to visual stimulation. Imaging was conducted on a clinical MRI scanner using blood-oxygen-level-dependent (BOLD) contrast. Visual stimuli were delivered monocularly to the treated eye during image acquisition, with the contralateral eye occluded. Stimulus presentation was synchronised with image acquisition. Block-design visual paradigms were presented using an NNL Aktiva fMRI interface (Nordic NeuroLab, Bergen, Norway). Each paradigm lasted approximately 5 minutes, and testing was performed for each eye separately at baseline, and repeated at 2 days, 14 days, and 28 days following treatment. When an eye subsequently received a higher dose, a new baseline scan was acquired approximately 3 months after the initial treatment. Stimulus paradigms were selected according to baseline visual function. For Cohort 1 participants (NLP/BLP), paradigms included full visual field on/off stimulation, switching hemifield stimulation, and flickering checkerboard presentations. For Cohort 2 participants (hand movements/count fingers), paradigms included E-down, moving line, and flickering checkerboard presentations. Functional images were acquired using standard echo-planar imaging sequences.

Preprocessing steps included motion correction, spatial normalisation to a standard brain template, and spatial smoothing. Regions of interest encompassing primary and extrastriate visual cortex were defined anatomically. fMRI data were reviewed descriptively to assess stimulus-evoked cortical signal patterns. Image review and interpretation were performed by a single

neuroradiologist, blinded to visit order and dose level, using Siemens (Erlangen, Germany) Syngo Via workstation software with post-processing using NordicNeuroLab (Bergen, Norway) software and predefined manual thresholding procedures.

Earplug protection was provided to all participants, and padded head clamps were applied to minimise head motion. T1-weighted anatomical images were acquired using a three-dimensional magnetisation-prepared rapid acquisition gradient echo sequence (1 slab; 176 sagittal slices; field of view, 256 × 256 mm; slice thickness, 1 mm; isotropic voxel size, 1 × 1 × 1 mm; repetition time, 2300 ms; echo time, 2.98 ms; inversion time, 900 ms; flip angle, 9°).

BOLD fMRI data were acquired using a gradient-echo, simultaneous multi-slice accelerated echo-planar imaging T2*-weighted sequence (54 transversal slices angled to avoid the maxillary sinuses; in-plane resolution, 2 × 2 mm; slice thickness, 2.5 mm; field of view, 192 × 192 mm; repetition time, 3000 ms; echo time, 30 ms; flip angle, 90°; echo spacing, 0.65 ms; bandwidth, 1774 Hz/Px; total acceleration factor of 4 [SMS 2, GRAPPA 2]). One hundred and ten measurements were acquired for each paradigm, except for the flickering checkerboard paradigm, for which 90 measurements were acquired.

Field maps were acquired using a dual-echo gradient-echo pulse sequence (36 slices aligned to fMRI acquisition; slice thickness, 3 mm without gap; voxel size, 3 × 3 × 3 mm; field of view, 192 × 192 mm; repetition time, 400 ms; echo times, 4.92/7.38 ms; flip angle, 60°).

Supplementary Video 1: Participant-reported light perception following KIO-301 administration.

Post-study interview with a participant with longstanding no light perception who described the onset of light perception within days of intravitreal KIO-301 administration. This video reflects participant-reported experience and is provided for illustrative purposes.
