# Supplementary material for: Intravitreal photoswitch therapy in advanced retinitis pigmentosa: a phase 1 open-label trial
Source: Nat Med. 2026 Apr 14;32(5):1865–70. doi: 10.1038/s41591-026-04317-6 (PMC13190265; doi:10.1038/s41591-026-04317-6)
Supplement: Supplementary file 2 — Reporting Summary [file 41591_2026_4317_MOESM2_ESM.pdf]

nature portfolio

Corresponding author(s): Robert J. Casson

Last updated by author(s): Feb 8, 2026

Reporting Summary

Nature Portfolio wishes to improve the reproducibility of the work that we publish. This form provides structure for consistency and transparency in reporting. For further information on Nature Portfolio policies, see our Editorial Policies and the Editorial Policy Checklist.

Statistics

For all statistical analyses, confirm that the following items are present in the figure legend, table legend, main text, or Methods section.

n/a	Confirmed	
☐	☒	The exact sample size (*n*) for each experimental group/condition, given as a discrete number and unit of measurement
☐	☒	A statement on whether measurements were taken from distinct samples or whether the same sample was measured repeatedly
☒	☐	The statistical test(s) used AND whether they are one- or two-sided<br>*Only common tests should be described solely by name; describe more complex techniques in the Methods section.*
☒	☐	A description of all covariates tested
☒	☐	A description of any assumptions or corrections, such as tests of normality and adjustment for multiple comparisons
☒	☐	A full description of the statistical parameters including central tendency (e.g. means) or other basic estimates (e.g. regression coefficient) AND variation (e.g. standard deviation) or associated estimates of uncertainty (e.g. confidence intervals)
☒	☐	For null hypothesis testing, the test statistic (e.g. *F*, *t*, *r*) with confidence intervals, effect sizes, degrees of freedom and *P* value noted<br>*Give P values as exact values whenever suitable.*
☒	☐	For Bayesian analysis, information on the choice of priors and Markov chain Monte Carlo settings
☒	☐	For hierarchical and complex designs, identification of the appropriate level for tests and full reporting of outcomes
☒	☐	Estimates of effect sizes (e.g. Cohen's *d*, Pearson's *r*), indicating how they were calculated

Our web collection on statistics for biologists contains articles on many of the points above.

Software and code

Policy information about availability of computer code

Data collection	No custom code was used for data collection.
Data analysis	No custom software or algorithms were developed for this study. Functional MRI preprocessing, visualization and descriptive assessment were performed using standard, commercially available clinical software packages, as detailed in the Supplementary Methods.

For manuscripts utilizing custom algorithms or software that are central to the research but not yet described in published literature, software must be made available to editors and reviewers. We strongly encourage code deposition in a community repository (e.g. GitHub). See the Nature Portfolio guidelines for submitting code & software for further information.

Data

Policy information about availability of data

All manuscripts must include a data availability statement. This statement should provide the following information, where applicable:

- Accession codes, unique identifiers, or web links for publicly available datasets
- A description of any restrictions on data availability
- For clinical datasets or third party data, please ensure that the statement adheres to our policy

De-identified participant-level data supporting the findings of this study are not publicly available due to institutional ethics restrictions, sponsor confidentiality obligations, and the small sample size of this first-in-human clinical study, which may increase re-identification risk.
The minimum dataset necessary to interpret, verify and extend the findings reported in this Article is available from the corresponding author upon reasonable

request. Requests for access should be directed to the corresponding author (R.J.C.; email: robert.casson@adelaide.edu.au) and will be considered subject to institutional review board/ethics approval where required, sponsor review, and execution of an appropriate data-use/confidentiality agreement. Approved data may be used solely for non-commercial academic research consistent with the original informed consent and ethics approvals and may not be redistributed. Requests will be acknowledged within one week and a decision provided within two weeks.

Research involving human participants, their data, or biological material

Policy information about studies with human participants or human data. See also policy information about sex, gender (identity/presentation), and sexual orientation and race, ethnicity and racism.

Reporting on sex and gender	Sex was recorded as a biological attribute based on participant self-report. Gender identity was not specifically assessed. The study was conducted in accordance with the SAGER (Sex and Gender Equity in Research) guidelines; however, no sex- or gender-based analyses were performed due to the small sample size and the safety-focused, first-in-human Phase 1 design.
Reporting on race, ethnicity, or other socially relevant groupings	Race, ethnicity, or other socially constructed or socially relevant groupings were not collected or analysed in this study. Given the small sample size and the primary focus on safety in a first-in-human Phase 1 clinical trial, analyses stratified by race or ethnicity were not performed.
Population characteristics	Participants were adults with advanced retinitis pigmentosa and profound vision loss, as defined by the study inclusion criteria. Relevant population characteristics, including age, sex, diagnosis, disease severity, and prior treatments, are described in the manuscript. No genotypic stratification was performed, and the study was not powered to examine associations with demographic or clinical subgroups.
Recruitment	Participants were recruited from specialist retinal clinics according to predefined inclusion and exclusion criteria. As a first-in-human, safety-focused Phase 1 study in a rare disease population, recruitment was necessarily limited and may be subject to selection bias toward individuals willing and eligible to participate in an early-phase interventional trial. These factors may limit generalisability of the findings.
Ethics oversight	The study protocol was reviewed and approved by the Central Adelaide Local Health Network Human Research Ethics Committee and registered on ClinicalTrials.gov (NCT05282953). The study was conducted in accordance with the Declaration of Helsinki and the International Conference on Harmonisation Good Clinical Practice guidelines. All participants provided written informed consent prior to participation. Full details of ethics approval are provided in the manuscript.

Note that full information on the approval of the study protocol must also be provided in the manuscript.

Field-specific reporting

Please select the one below that is the best fit for your research. If you are not sure, read the appropriate sections before making your selection.

☒ Life sciences ☐ Behavioural & social sciences ☐ Ecological, evolutionary & environmental sciences

For a reference copy of the document with all sections, see nature.com/documents/nr-reporting-summary-flat.pdf

Life sciences study design

All studies must disclose on these points even when the disclosure is negative.

Sample size	Twelve eyes from six participants were included. Sample size was determined by the exploratory, safety-focused design of this first-in-human Phase 1 clinical study and was not based on a formal statistical power calculation.
Data exclusions	No data were excluded from the analyses.
Replication	This was a first-in-human clinical study, and findings were not replicated in independent cohorts.
Randomization	Not randomized. This was an open-label, non-randomized, safety-focused Phase 1 clinical study.
Blinding	Not blinded. Given the open-label design and primary focus on safety, neither participants nor investigators were masked to the intervention.

Reporting for specific materials, systems and methods

We require information from authors about some types of materials, experimental systems and methods used in many studies. Here, indicate whether each material, system or method listed is relevant to your study. If you are not sure if a list item applies to your research, read the appropriate section before selecting a response.

Materials & experimental systems

n/a	Involved in the study
☒	☐ Antibodies
☒	☐ Eukaryotic cell lines
☒	☐ Palaeontology and archaeology
☐	☒ Animals and other organisms
☐	☒ Clinical data
☒	☐ Dual use research of concern
☒	☐ Plants

Methods

n/a	Involved in the study
☒	☐ ChIP-seq
☒	☐ Flow cytometry
☐	☒ MRI-based neuroimaging

Animals and other research organisms

Policy information about studies involving animals; ARRIVE guidelines recommended for reporting animal research, and Sex and Gender in Research

Laboratory animals	Retinal explants were obtained from rd1 mice (retinal degeneration type 1) aged postnatal day 30–60. Animals were housed under standard laboratory conditions on a 12:12 h light–dark cycle with controlled temperature (21 °C) and humidity.
Wild animals	This study did not involve wild animals.
Reporting on sex	The sex of mice was not a variable in the preclinical validation experiments and was not considered in the study design or analysis. Experiments were performed on retinal explants to confirm biological activity of the compound prior to clinical dosing. No sex-based analyses were performed.
Field-collected samples	This study did not involve field-collected samples.
Ethics oversight	All animal procedures were approved by the University of California, Berkeley Institutional Animal Care and Use Committee (protocol AUP-2016-04-8700-3) and conducted in accordance with the NIH Guide for the Care and Use of Laboratory Animals.

Note that full information on the approval of the study protocol must also be provided in the manuscript.

Clinical data

Policy information about clinical studies

All manuscripts should comply with the ICMJE guidelines for publication of clinical research and a completed CONSORT checklist must be included with all submissions.

Clinical trial registration	This investigator-initiated, first-in-human Phase 1 study was registered at ClinicalTrials.gov (NCT05282953) prior to enrolment.
Study protocol	The full clinical protocol is not publicly posted owing to sponsor-confidential information related to investigational compound formulation and dosing strategy. A redacted version of the protocol sufficient to understand study design and conduct is available from the corresponding author upon reasonable request for academic purposes, subject to institutional and sponsor review and appropriate data-sharing agreements.
Data collection	Participants with advanced retinitis pigmentosa were recruited and followed prospectively between November 2022 and September 2023. The first participant provided consent on 3 November 2022 and the last participant provided consent on 27 March 2023. Follow-up for the final participant was completed on 8 September 2023.<br>Clinical study visits, treatment administration and safety assessments were conducted at the Royal Adelaide Hospital (Adelaide, Australia) and the Harley Eye Clinic (Adelaide, Australia). Functional vision, light perception and visual field assessments were performed in a dedicated clinical research facility in Adelaide, Australia, operated by the study sponsor. Functional MRI assessments were conducted at the South Australian Health and Medical Research Institute (SAHMRI), Adelaide, Australia. Bioanalytical sample processing was performed at Agilex Biolabs Pty Ltd (Thebarton, Australia).<br>All data were collected prospectively according to the protocol using standardized clinical, safety and exploratory functional outcome measures at scheduled study visits.
Outcomes	The primary objective of the study was to assess the safety and tolerability of intravitreal KIO-301 administration. Safety outcomes were pre-specified in the protocol and included the incidence and severity of adverse events, serious adverse events, and ophthalmic examination findings over the follow-up period. Secondary and exploratory outcomes included functional and imaging measures intended to assess potential biological activity and target engagement. Given the first-in-human, small-sample design, all non-safety outcomes were pre-specified as exploratory and were summarized descriptively without formal hypothesis testing.

Plants

Seed stocks	No plant materials, seed stocks or plant-derived biological materials were used in this study.
Novel plant genotypes	No plant genotypes, transgenic plant lines or gene-edited plant materials were generated or used.
Authentication	No plant materials were used; therefore, no plant authentication procedures were required.

Magnetic resonance imaging

Experimental design

Design type	Task-based (visual stimulation), block design (BOLD fMRI).
Design specifications	Monocular visual stimulation was delivered during BOLD fMRI acquisition (treated eye stimulated; contralateral eye occluded), with stimulus presentation synchronized to image acquisition. Block-design paradigms were presented using an NNL Aktiva fMRI interface (Nordic NeuroLab). Each paradigm lasted ~5 minutes; 110 volumes were acquired for each paradigm (90 volumes for the flickering checkerboard paradigm). Testing was performed for each eye separately at baseline and repeated at 2, 14 and 28 days post-treatment; when an eye later received a higher dose, a new baseline scan was acquired ~3 months after initial treatment.
Behavioral performance measures	No in-scanner behavioral responses were collected (no button-press/response-time outcomes). The fMRI component assessed stimulus-evoked BOLD signal changes during passive viewing of monocular visual stimuli.

Acquisition

Imaging type(s)	Functional MRI: task-based BOLD fMRI (T2*-weighted EPI).<br>Structural MRI: T1-weighted 3D MPRAGE.<br>Field mapping: dual-echo gradient-echo field maps.
Field strength	Clinical MRI scanner (Siemens platform). Field strength: 3 Tesla.
Sequence & imaging parameters	T1-weighted anatomical (3D MPRAGE): 176 sagittal slices; FOV 256 × 256 mm; slice thickness 1 mm; isotropic voxel 1 × 1 × 1 mm; TR 2300 ms; TE 2.98 ms; TI 900 ms; flip angle 9°.<br>BOLD fMRI (gradient-echo SMS-accelerated EPI, T2*): 54 transversal slices (angled to avoid maxillary sinuses); in-plane resolution 2 × 2 mm; slice thickness 2.5 mm; FOV 192 × 192 mm; TR 3000 ms; TE 30 ms; flip angle 90°; echo spacing 0.65 ms; bandwidth 1774 Hz/Px; acceleration factor 4 (SMS 2, GRAPPA 2); 110 measurements per paradigm (90 for flickering checkerboard).<br>Field maps (dual-echo GRE): 36 slices aligned to fMRI; slice thickness 3 mm (no gap); voxel 3 × 3 × 3 mm; FOV 192 × 192 mm; TR 400 ms; TE 4.92/7.38 ms; flip angle 60°.
Area of acquisition	Whole-brain acquisition with coverage including occipital visual cortex; EPI slices were acquired in a transversal orientation (angled to avoid maxillary sinuses).

Diffusion MRI ☐ Used ☒ Not used

Preprocessing

Preprocessing software	Preprocessing used Siemens Syngo Via workstation software. Preprocessing included motion correction, spatial normalization standard template and spatial smoothing with visual inspection of artifact and signal dropout. These procedures were applied consistently across all datasets. Analyses were conducted to support exploratory evaluation of stimulus-evoked BOLD responses in this first-in-human study (Supplementary Methods)..
Normalization	Functional MRI data were evaluated primarily in native subject space. Where spatial normalization was applied for visualization and exploratory modelling, alignment to a standard reference template was performed using standard routines.
Normalization template	Standard reference template space was used for visualization and exploratory modelling where required; primary evaluation was conducted in native subject space.
Noise and artifact removal	Standard preprocessing steps included motion correction, masking and visual inspection for motion-related and scanner-related artifacts. Datasets were reviewed for excessive motion or signal dropout prior to analysis.
Volume censoring	Data were visually inspected for motion and artifacts prior to inclusion in exploratory analyses. No formal volume scrubbing procedures were applied.

Statistical modeling & inference

Model type and settings	General linear modelling approaches were used to explore stimulus-evoked BOLD responses within individuals. Given the small sample size and first-in-human design, results are presented descriptively and were not used to support formal efficacy inference.
Effect(s) tested	Exploratory changes in stimulus-evoked BOLD signal over time within individuals.

Specify type of analysis: ☐ Whole brain ☒ ROI-based ☐ Both

Anatomical location(s)	BOLD fMRI data were acquired. Exploratory analyses focused on the occipital visual cortex. Regions of interest were defined anatomically based on structural MRI and standard neuroanatomical boundaries after normalization to standard space, with slices through the visual cortex shown for visualization of stimulus-evoked activity.
Statistic type for inference (See Eklund et al. 2016)	Given the first-in-human design and small sample size, analyses were exploratory and descriptive, and were not used to support formal group-level voxel-wise or cluster-wise statistical inference.
Correction	No formal multiple-comparison–corrected group-level statistical inference is reported in the manuscript. Modelling was performed for exploratory visualization and assessment of stimulus-evoked BOLD signal changes

Models & analysis

n/a	Involved in the study
☒	☐ Functional and/or effective connectivity
☒	☐ Graph analysis
☒	☐ Multivariate modeling or predictive analysis
